# Notes and News

**Published:** 1888-06-30

**Authors:** 


### Notes and News.

Collected and Communicated.

It is proposed to erect a cottage hospital at Ardrossan, N. B., and "A Summer Visitor," writing to the *Ayrshire Weekly News*, points out, that instead of going to the expense of making a new building it would be better to buy the Ardeer Assembly Rooms, which could be had cheaply and converted into a hospital with comparatively little trouble. Besides these advantages the Assembly Rooms are situated near the harbour, the Eglinton coal pits, and Nobel's explosives works, where accidents of various kinds are most likely to occur. This is no trifling consideration, and the good folk of Ardrossan will do well to consider their "Summer Visitor's" sensible and economical suggestion.

A much-needed addition has been made to the Royal Portsmouth, Portsea, and Gosport Hospital at Landport, which now contains 104 beds instead of 72 as formerly. The new wards were opened on May 30th by the Bishop of Winchester, in presence of General Sir George and Lady Willis, Admiral Sir George Willes, Sir William King, chairman of the Committee of Management, and a large company. Sir William King complained that the subscriptions to the hospital had not increased in due proportion to the population; but Sir George Willes protested that the people of the district were very generous if they were properly appealed to. It is to be hoped that the addition to the hospital, standing out against the main building that is weather-stained with years of noble service, will strike the inhabitants of Portsmouth and its neighbourhood as an eloquent though silent appeal.

The Lockhart Hospital at Lanark owes its existence to Sir Simon and Lady Macdonald Lockhart, of Lee, who first obtained possession and fitted up an old house that had once been used for cholera and subsequently for fever patients, and ten years ago erected a pretty and well-arranged hospital, where 107 in-patients and 230 out-patients have been treated during the last twelve months. It is interesting to note that, while the in-patients are rather fewer now than they were when the hospital opened —107 in 1887, against 115 in 1878— the number of out-patients has more than doubled, growing from 110 to 230. We see that the pay system is practised to some extent in the Lockhart Hospital, the patients' payments during last year having amounted to £17 19*s.* 2*d.* Lady and the Misses Macdonald still take a deep and kindly interest in the hospital, often sending gifts of books, clothing, and dainties for festival seasons, and Mrs. Thomas Somerville acts as honorary lady superintendent, while the active duties of the post of matron are in the efficient hands of Miss McRae, who has held that position since the hospital began its work.

It is not only English hospitals which have been receiving lucky windfalls of late. The governor of the public hospital at Mulhouse recently received an intimation that the executors of the late M. Emile Hübner, an Alsatian, were prepared to hand over for the use of the hospital a legacy of £20,000. The name of the late M. Hübner is well known in the City of London, and he has left several smaller sums to English charities.

The Society for the Employment of Women, 22, Berners Street, has formulated a scheme for sending working women out of town into the country as fruit-pickers and packers. The wages paid for the gathering average 12*s.* a week, and lodgings, under supervision, can be arranged at 8*s.* a week. Mr. Talleman, who read an important series of papers at the Bakers' Hall last winter on the British food supply, conceived the idea, which certainly might afford many a London sempstress or factory-hand with both change of labour and air in a reasonable and healthy manner.

On the 15th inst., at the Vegetarian Hotel, Buckingham Street, Strand, there was a public dinner and discussion in the interests of a proposed new hospital to be conducted on remarkable lines. In the general treatment of disease, all drugs are to be excluded. For means of cure, reliance is placed solely on diet and general hygienic treatment, such as bathing, massage, and so on. The diet will exclude all animal food. This new hospital is to be under the management of Dr. Allinson, of Duke Street, Portland Place, who, it seems, has for some years renounced drugs in his practice, and to whose success in the pursuit of health by carefully-devised living many people testified. It was announced that one subscriber has guaranteed £200 towards the institution, and there appears to be little doubt that the money needed for a modest beginning will be secured. The experiment will be watched with interest.

It is to be regretted that the Home for Invalid Children, Montpelier-road, Brighton—which the promotors claim to be the first convalescent home for children ever established—should be harassed by any lack of funds in continuing the good work it has been carrying on for the last thirty-two years. The terms asked—eight shillings a-week for each child—do not suffice to pay the expenses of such an institution, and the subscriptions and donations are so far from being sufficient to make up the deficit that, in spite of a special donation of £50 from the dividends of the Endowment Fund, the home ended the year with a debt of £61 14*s.* 8*d.* This is an institution which ought to be supported, not only by the inhabitants of Brighton, but by all who go there in search of health and pleasure, who would do well to remember how precious the sunny bracing air of "London by the Sea" must be to town children recovering from illness.

The Convalescent Home at Heytesbury, Wilts, the second annual report of which we have received from the lady superintendent, Miss Margaret Elling, is doing a thoroughly good work at a very moderate expense. During the twelve months between May, 1887, and May, 1888, fifty-four women, young and old, were received and cared for there, at a total cost of little more than £120. Some of these were recovering from severe illnesses and operations, but the great majority were suffering from anæmia, debility, and similar diseases, which demand for their chief treatment good air, good food, and perfect rest, the very things that convalescent homes can best supply. The Home being of small size is of great advantage to many of the patients, as it takes away the distressing feeling that it is an "institution," and as the lady superintendent has the happy faculty of winning their respect, and the inhabitants of the village, gentle and simple, show their interest by kindly gifts.

The following vacancies are announced this week, the amount of salary in each case being given after the name of the institution :—

**Resident Medical Officers.**

Chelsea Hospital for Women, Fulham Road, S.W. Resident Medical Officer.—£60 per annum, with board.
City of London Hospital for Diseases of the Chest, Victoria Park, E. Resident Clinical Assistant.
City of London Lunatic Asylum, Stone, near Dartford, Kent. *Locum tenens*.—Two guineas a week, with board, &c.
Doncaster General Infirmary and Dispensary. House Surgeon.—£100 per annum, with board.
Dundee Royal Infirmary. Resident Assistant House Surgeon.—£60 per annum.
East Suffolk and Ipswich Hospital. House Surgeon.—£80 per annum, with board.
Glasgow Hospital for Sick Children. Assistant House Surgeon.
Kent County Asylum, Barming Heath, Maidstone. Third Assistant Medical Officer.—£120 per annum.
West Sussex, East Hants, and Chichester Infirmary. House Surgeon.—£100, with board.
Worcester General Infirmary. House Surgeon.—£100 per annum, with board.

**Non-resident Medical Officers.**

Borough of Bradford. Medical Officer of Health.—£500 per annum.
City of London Hospital for Diseases of the Chest, Victoria Park, E.—Assistant Physician. Candidate must be Member or Fellow of the Royal College of Physicians, London.
City of London Hospital for Diseases of the Chest, Victoria Park, E. Pathologist.—£105 per annum.
Parish of Eddrachillis, Sutherland.—£150 per annum, with free house.
Parish of Kirkmabreck, Kirkcudbrightshire. Medical Officer for the Poor.—£35 per annum.
Parochial Board of Pennygorm and Torosay. Medical Officer.—£100 per annum.

---

The Sheffield guardians have unanimously voted £25 to Dr. G. P. Godfrey, medical officer to the workhouse, for extra services during the epidemic of small-pox.

---

Ordinary charity collections will look small beside the result of the centenary festival of the Royal Masonic Institution for Girls at which H.R.H. the Prince of Wales presided on the 7th inst.; and in proposing the toast of "Success to the Institution," mentioned that in 1838, at the celebration of the jubilee of the institution £1,000 was subscribed, and at the festival of 1871 £5,200 was collected. The result of the festival, however, was the very large sum of £50,500, which the Prince proudly held forth to the world as beating the record of charity dinners.

---

The annual meeting of the Hamilton Assocation for Providing Trained Male Nurses was held on Wednesday, June 20th, at the Grosvenor Hotel, Park Street, Grosvenor Square. Archdeacon B. F. Smith occupied the chair, and in moving the adoption of the report, described the progress which had been made during the year. The work done by the society had nearly doubled, as was shown by the amount received as wages for nurses having increased from £800 to £1,400 in a single year. The institution was not, however, as yet self-supporting, the loss on the working during the past year having amounted to £243 0*s*. 10*d*. An entirely new departure had been taken by providing trained male nurses gratuitously for the sick poor. Only a beginning had as yet been made in this direction, but it was proposed to extend the work as funds permitted. As yet the payments in aid of this branch had been made from the general fund, but in view of its continuance and extension a special fund was being raised to support it, and the Chairman announced that twenty pounds had already been received. The association continued to supply St. George's Hospital with male nurses, and during the past few months the Seamen's Hospital and Guy's Hospital had each obtained a man from the association. The services rendered by the nurses had in general given the greatest satisfaction, and the committee of the association may justly feel that it fills a valuable and unique place among nursing institutions.

The Memorial Cottage Hospital, Mildmay Park, N., exists in connexion with the Mildmay Mission, and is intended for the reception of the sick poor from the missions worked by the deaconesses. The hospital contains ten beds for men, ten for women, and seven cots for children. Most of these are fully endowed, and therefore permanently at the service of the sick poor, but funds are still needed to complete the endowment of the whole hospital, and even though it was considerably enlarged the beds could be suitably filled. A very pleasing feature which we note in the list of donations, is the sums given by nurses and collected by them for the endowment of the institution. It is a great testimony to the work of the hospital when its nurses are ready to subscribe to its funds.

---

Professor Biny, of Bonn, whose speech at the Vienna Congress seven years ago made such a sensation, has again been speaking on the subject of alcohol at Wiesbaden. Everything that can be said in favour of alcohol, according to Professor Biny, concerns invalids alone. The healthy need no stimulant for the heart, lungs, or stomach; the sufficiently nourished need no reparation for the waste of albumen; the non-feverish need no sedative to reduce internal heat. When the healthy man partakes of alcohol in any form, it can only be described as enjoyment, or as refreshment after lengthened bodily or mental exertion. The flooding of stomach and brain with beer by German students was severely condemned by Professor Biny; but his most amusing remarks were those on the temperance movement in England. Naturally he highly approved of it, but said that its spread, though partly due to increased knowledge of health laws, was chiefly due to the greater quantity of adulterated, and thereby highly alcoholized wine and brandy that is sold here; taken for pleasure it makes the healthy ill, and therefore could scarcely make the sick well when taken as medicine. In the discussion which followed the Professor's speech, it was generally acknowledged that the use of alcohol was liable to lead to its abuse, and that the word of warning might well be passed on to the people by the assembled physicians.

---

On Sunday, the 10th, the Rev. J. Hiles Hitchens, D.D., preached at the Eccleston Square Church, Belgrave Road, on behalf of the hospitals and dispensaries. He chose as his text Psalm xxv. 18, "Look upon my affliction and my pain." The preacher commenced by saying: "It was Arthur Hallam who once said, 'Pain is the deepest thing in our nature.' Certainly it is a mystery to all men, and its causes often perplex even the skilled physiologist. But there can no man be found who has not experienced pain and witnessed its terrible effects." He then proceeded to show that pain is not an unmixed evil; that dark, ugly, and dreadful as it is, still "a bright, phosphorescent light plays athwart it"; that our merciful God had done very much to alleviate, to sanctify, and to abolish pain, much to whisper hope, beget patience, and so turn the curse into a blessing. The points to which Dr. Hitchens specially called his hearers' attention were the following: God has provided remedies and anodynes; has converted pain into a medium of knowledge; has made pain instrumental in developing many personal and social excellencies; has caused the recollection of pain speedily to fade from our minds; has frequently sanctified pain to the spiritual and eternal welfare of the sufferer; and has disclosed a land where He has promised pain shall no more exist. From the working out of these thoughts the preacher drew two practical lessons. First, that even amid our pains we should be grateful to God and implicitly trust Him. Second, that we should imitate God. It is God-like to relieve pain, mitigate woe, banish wretchedness, and dry tears of sadness. Having quoted some statistics from the special number of The Hospital, giving the number of patients treated in the neighbouring hospitals during the past year and the state of the funds, Dr. Hitchens closed by an earnest appeal for generous help to these noble institutions.

